# Formulation Development of High Strength Gel System and Evaluation on Profile Control Performance for High Salinity and Low Permeability Fractured Reservoir

**DOI:** 10.1155/2017/2319457

**Published:** 2017-05-16

**Authors:** Chengli Zhang, Guodong Qu, Guoliang Song

**Affiliations:** ^1^School of Petroleum Engineering, Northeast Petroleum University, Daqing 163318, China; ^2^School of Mathematics and Statistics, Northeast Petroleum University, Daqing 163318, China

## Abstract

For the large pores and cracks of reservoirs with low temperatures, high salinity, and low permeability, a new type of high strength gel ABP system is developed in this paper. The defects of conventional gels such as weak gel strength, no gelling, and easy dehydration are overcome under the conditions of low temperature and high salinity. The temperature and salt resistance, plugging characteristics, and EOR of the gel system are studied. Under the condition of 32°C and 29500 mg/L salinity, the ABP system formulation is for 0.3% crosslinking agent A + 0.09% coagulant B + 3500 mg/L polymer solution P. The results show that when the temperature was increased, the delayed crosslinking time of the system was shortened and the gel strength was increased. The good plugging characteristics of the ABP system were reached, and the plugging rate was greater than 99% in cores with different permeability. A good profile control performance was achieved, and the recovery rate was improved by 19.27% on the basis of water flooding. In the practical application of the gel system, the salinity of formation water and the permeability of fractures are necessary to determine the appropriate formulation.

## 1. Introduction

With the rapid development of the economy, oil demand has increased greatly, and the exploration and development of the oilfield are becoming more and more complex [1]. The low permeability reservoir will be the major object of development in the future [2]. A large number of fractures often exist in low permeability reservoirs [3]. Water channeling, water submerging, and so on are often caused by water flooding in the development process [4]. So water shutoff and profile control measures need to be applied to the oil and water wells, and then the oil recovery of low permeability reservoirs is further improved [5–8]. The polymer gel system has been successfully used in some cases, such as in reservoirs with natural fractures [9], water-coning situations [10], carbonate reservoirs [11], open-hole horizontal wells [12], multizone wells [13], gas shutoff in an open-hole gravel pack [14], and acting as annular barriers [15]. Seright and Martin explained the effects of pH, rock permeability, and lithology on the performance of a resorcinol/formaldehyde gel [16]. The traditional single crosslinking system was reacted with polyacrylamide solution to form a gel, which is unfavorably applied in the field due to the single characteristics and many other restrictions [17, 18]. A water shutoff gel system formed with polyacrylamide and polyamine crosslinking agent, which provided sufficient gel times at less than 80°F, was presented by Reddy et al. [19]. Though a good plugging effect was reached by the SMA gel system, it was only suitable for low salinity (about 4000 mg/L) [20]. A formaldehyde phenolic resin prepolymer as high strength gel system was easily dehydrated under the condition of high salinity water, and, at a low temperature (less than 60°C), weak strength gel, or even no gel, may be formed by the viscoelastic polymer system [21].

JY oilfield located in the northwest of China belongs to the low permeability oilfields with low temperature and high salinity, with an average temperature, salinity, and permeability of 32°C, 29500 mg/L, and 10 mD. The development degree of primary fracture is medium. The width and permeability of fractures are, respectively, about 0.02 mm~20 mm and 300 mD~1000 mD. And a conventional system was difficult to use to implement profile control and water shutoff because of poor adaptability and short valid period. In this paper, according to the characteristics of JY oilfield, a high strength gel formulation of ABP system is developed suitable for reservoirs with low temperature, high salinity, and low permeability. Its performance was evaluated, and the disadvantages of the single system and traditional profile control agent were overcome.

## 2. Experimental Materials and Methods

The different concentrations of the crosslinking agent A (0.2%, 0.3%, and 0.4%) and coagulant B (0.05%, 0.09%, and 0.13%) were prepared in a laboratory. The crosslinking agent A is a cationic sulfonated phenolic resin, and its chemical structural formula is as follows:


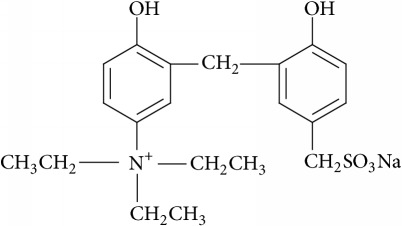


The coagulant B is phenolic resin produced by the condensation reaction of formaldehyde and phenol, and its chemical structural formula is as follows:


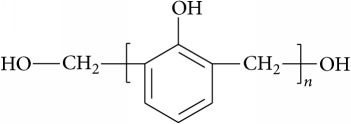


The polymer P was partially hydrolyzed polyacrylamide (HPAM), and the relative molecular weight was 25 million. According to the experimental schemes and requirements, the polymer solution of different concentrations (1500 mg/L, 2500 mg/L, and 3500 mg/L) was formulated.

The simulated formation oil of JY oilfield was used in the following experiments, and the average viscosity at 32°C was 2.6 mPa·s after the dehydration and degassing. The simulated formation water of JY oilfield was used in the experiments, and its salinity was 15000 mg/L, 29500 mg/L, and 45000 mg/L, respectively.

For studying the fracture system of the low permeability reservoir, the high permeability cores are 300 mD, 500 mD, and 1000 mD, respectively, for simulating the fracture systems. The low permeability cores are 10 mD for simulating the low permeability formation.

An HW-4A type double thermostat was used to maintain the temperature of the high strength gel system. The viscosity of the high strength gel was measured by a Brookfield DV III ultra-type rotary viscometer. The solution was stirred evenly by a JJ-1 type electric mixer. The rock flow experiment device was applied to evaluate the sealing characteristics of the high strength gel system and conduct the oil displacement experiment.

## 3. Results and Discussions

### 3.1. Experiment  1: Determination of High Strength Gel Formulation in Low Temperature and High Salinity Water

#### 3.1.1. Experimental Procedure

1. The solution of polymer P, crosslinking agent A, and coagulant B were mixed in accordance with the ratio of the experimental schemes and stirred evenly.
2. The mixed solutions were placed in a thermostat preheated to 32°C.
3. The viscosity of the gel system was measured by the time steps.

#### 3.1.2. When the Concentration of Polymer P Was 1500 mg/L

**Figure 1:**
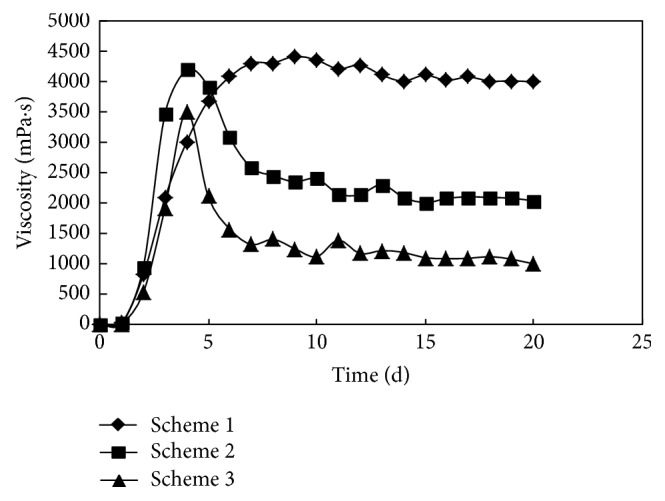
The changing process of viscosity when the concentration of polymer P was 1500 mg/L.

The initial viscosity of the gel system was 12.0 mP·s when the concentration of polymer P was 1500 mg/L. The three experimental schemes were designed under the conditions of different ratios of crosslinking agent A and coagulant B. The gelling process is shown in Figure 1.

*Scheme  1*. The concentrations of polymer P, crosslinking agent A, and coagulant B were 1500 mg/L, 0.2%, and 0.05%, respectively.

*Scheme  2*. The concentrations of polymer P, crosslinking agent A, and coagulant B were 1500 mg/L, 0.3%, and 0.09%, respectively.

*Scheme  3*. The concentrations of polymer P, crosslinking agent A, and coagulant B were 1500 mg/L, 0.4%, and 0.13%, respectively.

The initial viscosity of the system was low due to the concentration limits of the polymer solution. As a result of the electrostatic shield effect, polymer molecular chains were curled up significantly under the condition of high salinity, and the distance between molecules of the polymer was relatively enlarged [22]. Then the crosslinking agent acting as bridge could not function effectively. The characteristics of viscosity reduction were displayed obviously, and the gelling time was extended.

For schemes 2 and 3, due to the low polymer concentration and relatively high concentrations of crosslinking agent and coagulant, the initial viscosity of the gel system increased so fast that the viscosity reduction followed in a short time. The phenomenon of excessive crosslinking was evidenced by the shrinking of the net structure [23]. And then the water enclosed in the gel was squeezed out. The dehydration was exhibited in the early stages. It was indicated that the amount of coagulant and crosslinking agent should be moderate so that the phenomenon of excessive crosslinking would be avoided under a certain polymer concentration. In scheme 1, a slow downward trend of viscosity was displayed with time prolonging, and good viscosity stability was maintained. The gelling time was 6-7 days. The gel strength was well maintained, but the results were far from meeting the standard of high strength gel (viscosity > 10000 mPa·s).

#### 3.1.3. When the Concentration of Polymer P Was 2500 mg/L

**Figure 2:**
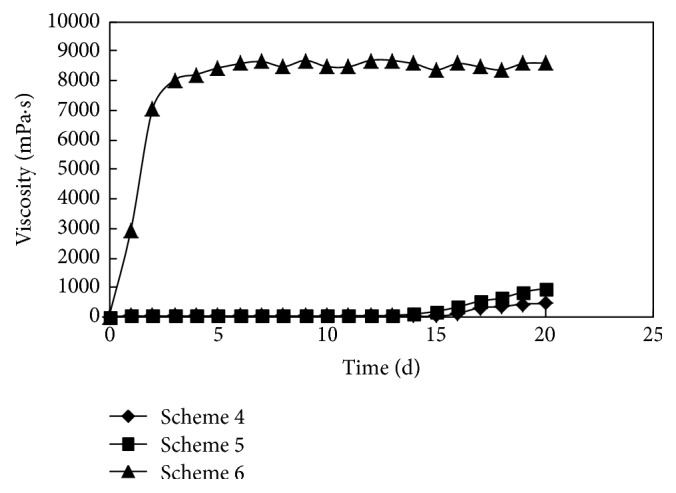
The changing process of viscosity when the concentration of polymer P was 2500 mg/L.

The initial viscosity was 22.6 mP·s when the concentration of polymer P was 2500 mg/L. The three experimental schemes were designed under the conditions of different ratios of crosslinking agent A and coagulant B. The gelling process is shown in Figure 2.

*Scheme  4*. The concentrations of polymer P, crosslinking agent A, and coagulant B were 2500 mg/L, 0.2%, and 0.09%, respectively.

*Scheme  5*. The concentrations of polymer P, crosslinking agent A, and coagulant B were 2500 mg/L, 0.3%, and 0.13%, respectively.

*Scheme  6*. The concentrations of polymer P, crosslinking agent A, and coagulant B were 2500 mg/L, 0.4%, and 0.05%, respectively.

For schemes 4 and 5, the viscosity of the system did not increase. The agglomeration and precipitation of coagulant occurred with that. The analysis showed that when the amount of coagulant was relatively greater, namely, that the ratio of crosslinking agent and coagulant was less than 3 : 1, the reaction activation energy of the crosslinking agent and coagulant molecular was reduced due to low temperature and a large number of divalent metal ions. The coagulant could not be reacted with crosslinking agent, there was no crosslinking for a long time, the coagulant was precipitated, and, ultimately, the crosslinking failed. Therefore, when the proportion of crosslinking agent and coagulant was less than 3 : 1, and low temperature and high salinity were presented at the same time, the high strength gel could not be formed. When the concentration of polymer P was 2500 mg/L, the 8500 mPa·s could be reached by adding moderate amount of crosslinking agent and coagulant, and the gelling time was 5-6 days. The gel strength was well maintained, but the standard of high strength gel (viscosity > 10000 mPa·s) was not achieved.

As can be seen from the experimental schemes 1–6, the delayed crosslinking time decreases with the increase of polymer concentration and the amount of crosslinking agent. The coagulant should not be used excessively, and the reasonable proportion of the crosslinking agent and coagulant should be more than 3 : 1. The gel stability increases with the polymer concentration, when the amounts of the crosslinking agent and coagulant increase, and the gel stability initially increases and then decreases. The greater the initial viscosity of the system is, the better the gelling effect is. Otherwise, the phenomenon of polymer molecular curling will be exhibited, intermolecular distances will be extended, and then crosslinking points of molecular will be decreased under the condition of high salinity.

#### 3.1.4. When the Concentration of Polymer P Was 3500 mg/L

**Figure 3:**
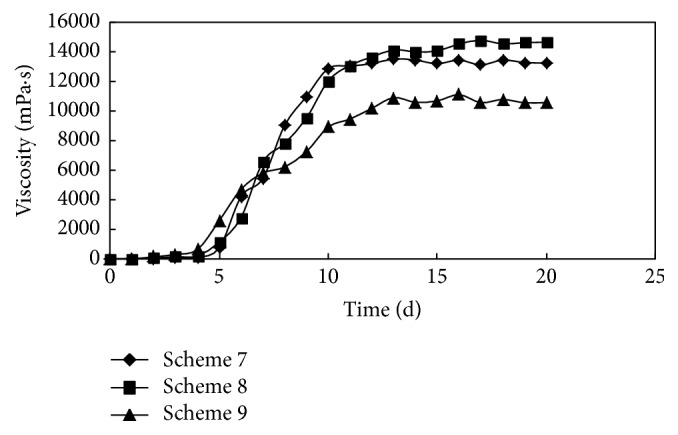
The changing process of viscosity when the concentration of polymer P was 3500 mg/L.

According to the above experimental analysis and results, schemes 7–9 were designed under the conditions that the ratio of crosslinking agent A and coagulant B is more than 3 : 1. The initial viscosity was 33.0 mPa·s when the concentration of polymer P was 3500 mg/L. The gelling process is shown in Figure 3.

*Scheme  4*. The concentrations of polymer P, crosslinking agent A, and coagulant B were 3500 mg/L, 0.2%, and 0.05%, respectively.

*Scheme  5*. The concentrations of polymer P, crosslinking agent A, and coagulant B were 3500 mg/L, 0.3%, and 0.09%, respectively.

*Scheme  6*. The concentrations of polymer P, crosslinking agent A and coagulant B were 3500 mg/L, 0.4%, and 0.13%, respectively.

When the concentration of polymer P was 3500 mg/L, and the proportion of the crosslinking agent A and coagulant B was more than 3 : 1, the standard of high strength gel could be achieved. The gelling time was prolonged, and the gel stability was well maintained. Compared with scheme 8, the dosages of the crosslinking agent A and coagulant B were slightly lower in scheme 7, and the gel strength was correspondingly lower than that of the scheme 8. The gel strength of scheme 8 was 14500 mPa·s and the highest in the 9 schemes. The slightly higher amounts of crosslinking agent A and coagulant B were used in scheme 9. The reaction activation energy decreased, so the gel strength was lower than that of schemes 7 or 8. The experimental results of different formulations under the conditions of 32°C and 29500 mg/L salinity are shown in Table 1.

The results can be obtained in summary of the experimental schemes 1–9:

1. The delayed crosslinking time decreases with the increase of polymer concentration and the amount of crosslinking agent A. Under a certain polymer concentration, the amounts of crosslinking agent A and coagulant B should be suitable so that the phenomenon of excessive crosslinking is avoided. From the point of delayed crosslinking time, the amount of crosslinking agent that should be used is less.
2. The gel stability increases with the polymer concentration, when the amounts of the crosslinking agent and coagulant are increased, and the gel stability initially increases and then decreases. The reasons why the viscosity decreases include that the gel is dehydrated by excessive crosslinking.
3. The greater the initial viscosity of the system is, the better the gelling effect is, and the reasonable initial viscosity should be more than 30 mPa·s. The greater the initial viscosity of the system is, the better the gelling effect is. Otherwise, the phenomenon of polymer molecular curling will be exhibited, intermolecular distances will be extended, and then crosslinking points of molecular will be decreased under the condition of high salinity.
4. The coagulant B should not be used excessively, and the reasonable proportion of the crosslinking agent and coagulant should be more than 3 : 1. It is an important basis provided for the selection of the ABP system formulation.

The high strength gel is applied to plug big channels and fractures. Under the conditions of 29500 mg/L salinity water, considering the gel strength, delayed crosslinking time, stability, and other factors, the optimized formulation is 0.3% crosslinking agent A + 0.09% coagulation B + 3500 mg/L polymer solution P. The initial viscosity is about 30 mPa·s, which is easily injected in early stages and then in favor of forming high strength gel (>10000 mPa·s). In the process of practical application, formulations should be selected on the basis of the salinity conditions.

### 3.2. Experiment  2: Evaluation of Salt Resistance

#### 3.2.1. Experimental Procedure

Under the conditions of different salinities (15000 mg/L, 29500 mg/L, and 45000 mg/L), the optimized high strength gel system was prepared and placed in the thermostat at 32°C. The viscosity of the gel system was measured by the time steps to evaluate the performance of the high strength gel.

#### 3.2.2. Results and Discussions

**Figure 4:**
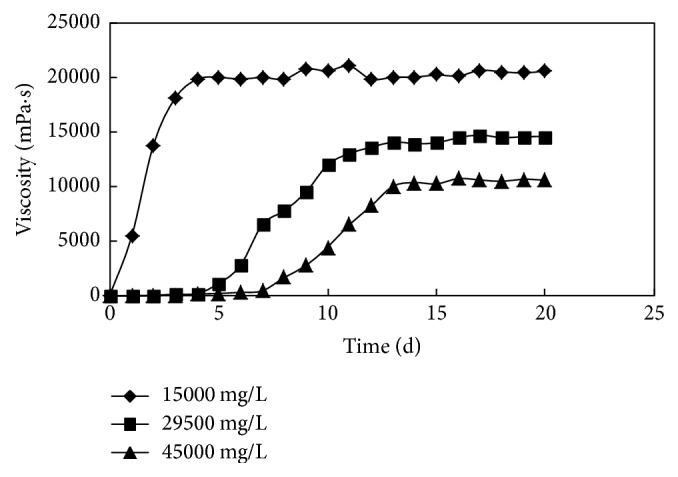
The changing process of viscosity at different salinities.

The results of the experiment are shown in Table 2 and Figure 4. With the increase of salinity, the polymer molecular chains were curled up significantly on the effect of electrostatic shield; the viscosity reduction of the polymer solution was obvious, so the initial viscosity of the system was low, and the distance between the polymer molecules was relatively greater; the crosslinking agent acting as a bridge for crosslinking particles could not function effectively. At the same time, under the action of a large number of divalent metal ions, the reaction of coagulant and crosslinking agent was hindered, the crosslinking activity was reduced, finally the gelation time of the system was prolonged, and the gel strength decreased. However, under the condition of high salinity of 45000 mg/L, the viscosity of the gel system could still meet the requirements of high strength gel, and the stability of the system was maintained for a long time.

### 3.3. Experiment  3: Evaluation of Temperature Resistance

#### 3.3.1. Experimental Procedure

The high strength gel systems were placed in the thermostat at different temperatures (20°C, 32°C, 45°C, and 60°C). The viscosity of the gel system was measured by the time steps to evaluate the performance of the gel.

#### 3.3.2. Results and Discussions

**Figure 5:**
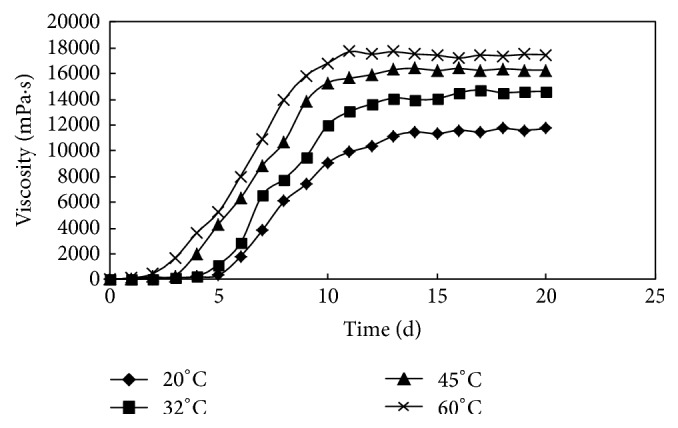
The changing process of viscosity at different temperatures.

The results of the experiment are shown in Table 3 and Figure 5. It is indicated that the curling degree of polymer molecular is reduced with the increase of temperature, resulting in the shortening of the intermolecular distance, the crosslinking agent A acts as a crosslinking bridge well, the multiples of hygroscopic expansion are increased, and then the net structure is further expanded [23]. Therefore, compared with the gel characteristics at 32°C, the lower the temperature is, the longer the gelling time is and the lower the gel strength is. On the contrary, the higher the temperature is, the shorter the gelling time is and the greater the gel strength is.

### 3.4. Experiment  4: Plugging Experiment

#### 3.4.1. Experimental Procedure

**Figure 6:**
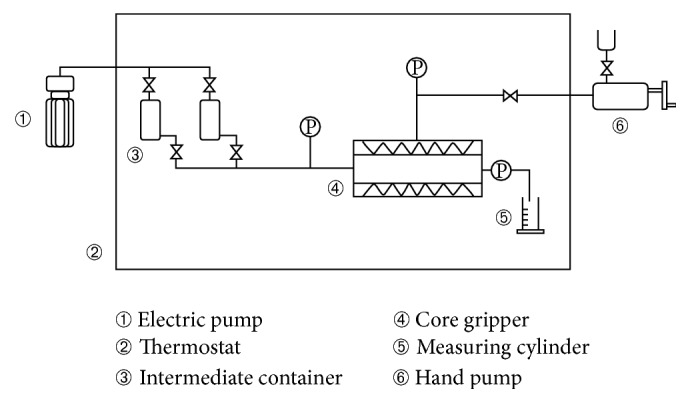
Core flow experiment device flowchart.

1. The cores were evacuated and saturated with the formation water (salinity 29500 mg/L) and then placed in the thermostat at 32°C for 8 hours.
2. The water flooding was carried out at the speed of 0.2 mL/min until the injection pressure was maintained stably. The stable pressure difference Δ*P*_1_ was recorded, and then the permeability to water *K*_1_ was calculated.
3. The ABP system was injected into the cores at the speed of 0.2 mL/min and was left to gel for 2–4 days.
4. The following water flooding was implemented at the speed of 0.2 mL/min, and the permeability to water *K*_2_ was calculated after the injection of the ABP system. The plugging rates were calculated by (*K*_1_ − *K*_2_)/*K*_1_ × 100%. The experimental flowchart is shown in Figure 6.

#### 3.4.2. Results and Discussions

Three cores with different permeability were used in the experiments, and the results are shown in Table 4.

As can be seen, after the ABP system is injected into cores of different permeability, good plugging effect is achieved. The plugging rate is up to 99.7% in 1000 mD core and 100% plugging rate could be reached in cores of permeability 300 mD and 500 mD.

### 3.5. Experiment  5: Oil Displacement Experiment

#### 3.5.1. Experimental Procedure

**Figure 7:**
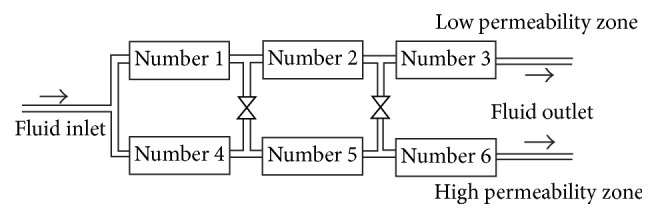
The series-parallel connection cores model sketch map.

1. The parallel cores model (cores numbers 1–6) was evacuated and saturated with the formation water (salinity 29500 mg/L) and then placed in the thermostat maintained at 32°C for 8 hours.
2. The model was saturated with crude oil. The oil saturation was calculated and then placed under the same condition for 8 hours.
3. Formation water was used to conduct the water flooding experiments at the speed of 0.20 mL/min. When the 70% water cut was reached in the high permeability zone, the first plugging was carried out, and the plugging depth was equivalent to 10 cm.
4. After the high strength gel was completely formed by the ABP system solution, the workflow was inserted to implement the water flooding. When the 90% water cut was reached in the high permeability zone, the second plugging was carried out, and the plugging depth was equivalent to 20 cm.
5. After the high strength gel was completely formed by the ABP system solution, the workflow was inserted to implement the water flooding. When the 90% water cut was reached in the high permeability zone in second time, the third plugging was carried out, and the plugging depth was equivalent to 30 cm.
6. After the high strength gel was completely formed by the ABP system solution, the workflow was inserted to implement the water flooding, until the 90% water cut was reached in the high permeability zone the third time. The amount of injection and production was recorded at each stage and the recovery rate was calculated. The series-parallel connection cores model is shown in Figure 7.

#### 3.5.2. Results and Discussions

**Figure 8:**
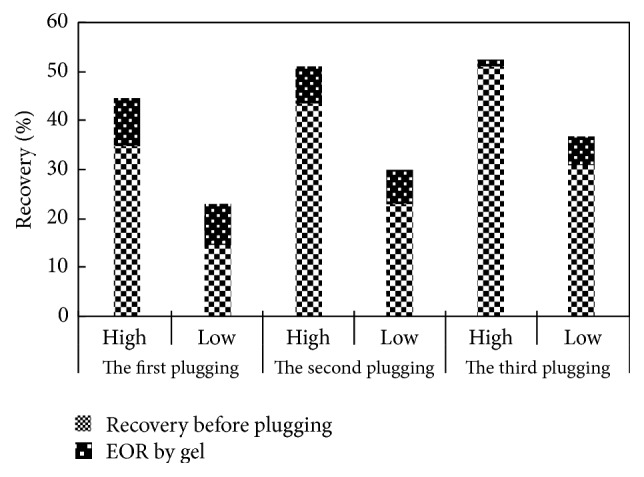
The sketch map of EOR by the ABP high strength gel.

The results of the depth profile control experiment of the optimized ABP high strength gel system are shown in Table 5 and Figure 8. In the initial water flooding process, the injection water was mainly pushed along the high permeability zone because the significant difference exists between the high and low permeability zone. When the 70% water cut was reached in the high permeability zone, the recovery of the high and low permeability zones was 34.62% and 14.42%, respectively, and the total recovery was 25.30%.

The first plugging was carried out in a high permeability zone (core number 4), namely, 10 cm profile control depth, and then the workflow was inserted to put the water flooding into effect. The high permeability zone (number 4) was bypassed and the oil in low permeability zone (number 1) was displaced by the injection water. The high permeability cores numbers 5 and 6 were displaced by injected water after completely flooding core number 1; the flooding area was enlarged, when 90% water cut was reached in the high permeability zone; almost no liquid flowed out from the low permeability zone. When the profile control depth was 10 cm, the recovery of high and low permeability zones were 44.30% and 22.81%, respectively. Compared with no plugging, the recovery of high and low permeability zone was, respectively, enhanced by 9.68% and 8.39%, and the total recovery was up to 34.39%, which was enhanced by 9.08%.

The second plugging was carried out in a high permeability zone, namely, 20 cm profile control depth (numbers 4 and 5), and then the water flooding workflow was inserted to put the water flooding into effect. The high permeability zone (numbers 4 and 5) was bypassed and the oil in low permeability zone (numbers 1 and 2) was displaced by the injection water. The high permeability core number 6 was displaced by injected water after completely flooding cores numbers 1 and 2. The flooding area was further enlarged. When the 90% water cut was reached in the high permeability zone, almost no liquid flowed out from the low permeability zone. When the profile control depth was 20 cm, the recovery of the high and low permeability zones was 51.83% and 29.74%, respectively. Compared with no plugging and the first plugging, the recovery of the high permeability zone was, respectively, enhanced by 17.21% and 7.53%, and that of the low permeability zone was, respectively, enhanced by 15.32% and 6.93%. The total recovery was 41.64%, which was, respectively, enhanced by 16.34% and 7.25%.

In the same way, when the profile control depth was up to 30 cm, the recovery of the high and low permeability zone was 53.20% and 35.58%, respectively. Compared with no plugging, the first plugging, and the second plugging, the recovery of the high permeability zone was, respectively, enhanced by 18.6%, 8.90%, and 1.37%, and the recovery of the low permeability zone was, respectively, enhanced by 21.16%, 12.77%, and 5.84%. The total recovery was 45.07%, which was, respectively, enhanced by 19.77%, 10.69%, and 3.43%.

From the above analysis, it is indicated that the injection water flooding area is enlarged after plugging high permeability channels and fractures, and the recovery is enhanced significantly. The deeper the depth is plugged, the greater the recovery is enhanced. Except for the fact that the recovery is enhanced in the low permeability zone, oil production is improved continuously in the high permeability zone; that is to say that the ABP plugging fluid is of some displacement effect on high permeability zone. The purpose of depth profile control is achieved by the ABP high strength gel, and a certain displacement effect is owned at the same time. Not only is the sweep extent improved in the low permeability zone, but also the residual oil is flooded in the high permeability zone, and then the oil recovery is enhanced.

## 4. Conclusions

1. The delayed crosslinking time decreases with the increase of polymer concentration and the amount of crosslinking agent A. Under a certain polymer concentration, the amount of crosslinking agent A and coagulant B should be suitable so that the phenomenon of excessive crosslinking is avoided. From the point of delayed crosslinking time, the amount of crosslinking agent should be used less. The coagulant B should not be used excessively, and the reasonable proportion of the crosslinking agent and coagulant should be more than 3 : 1.
2. The greater the initial viscosity of the system is, the better the effect on gelling is. The high strength gel is applied to plug big channels and fractures. The initial viscosity is about 30 mPa·s, which is easily injected in early stages and then in favor of forming high strength gel (>10000 mPa·s). Under the conditions of 29500 mg/L salinity water and 32°C, considering the gel strength, delayed crosslinking time, stability, and other factors, the optimized formulation is 0.3% crosslinking agent A + 0.09% coagulation B + 3500 mg/L polymer solution P. In the process of practical application, formulations should be appropriately selected according to the salinity conditions.
3. With the increase of salinity, under the action of the electrostatic shield and a large number of divalent metal ions, the polymer molecular chains were curled up seriously, and the reaction of coagulant and crosslinking agent was hindered. The crosslinking activity was reduced. Finally the gelation time of the system was prolonged, and the gel strength decreased.
4. The curling degree of polymer molecular is reduced with the increase of temperature, resulting in the fact that the intermolecular distance is relatively shortened. The crosslinking agent A acts as a crosslinking bridge as well. The multiples of hygroscopic expansion are increased, and then the net structure is further expanded. Compared with the gel characteristics at 32°C, the lower the temperature is, the longer the gelling time is and the lower the gel strength is. On the contrary, the higher the temperature is, the shorter the gelling time is and the greater the gel strength is.
5. The purpose of depth profile control is achieved by the ABP high strength gel, and the recovery is enhanced greatly with the plugging depth increasing. A certain displacement effect is owned at the same time. Not only is the sweep extent improved in the low permeability zone, but also the residual oil is flooded in the high permeability zone, and then the oil recovery is enhanced.

## Figures and Tables

**Table 1:** The experimental results of different ABP systems in 29500 mg/L salinity water.

Formulations				Delayed crosslinking time (days)	Gel strength (mPa·s)	Initial viscosity (mPa·s)	Stability (days)
Scheme number	Polymer P (mg/L)	Crosslinking agent A (%)	Coagulant B (%)				
1	1500	0.2	0.05	7-8	>4000	12.0	>30
2	1500	0.3	0.09	7-8	>2000	12.0	>30
3	1500	0.4	0.13	6-7	>1000	12.0	>30
4	2500	0.2	0.09	—	—	22.6	—
5	2500	0.3	0.13	—	—	22.6	—
6	2500	0.4	0.05	5-6	>8500	22.6	>60
7	3500	0.2	0.05	5-6	>13000	33.0	>60
8	3500	0.3	0.09	5-6	>14500	33.0	>60
9	3500	0.4	0.13	3-4	>10500	33.0	>60

**Table 2:** The gelling performance of the ABP system at different salinities.

Formulation					Delayed crosslinking time (days)	Gel strength (mPa·s)	Initial viscosity (mPa·s)	Stability (days)
Temperature (°C)	Polymer P (mg/L)	Crosslinking agent A (%)	Coagulant B (%)	Salinity (mg/L)				
32	3500	0.3	0.09	15000	2-3	>20000	110.7	>60
				29500	5-6	>14500	33.0	>60
				45000	7-8	>10500	21.2	>60

**Table 3:** The gelling performance of ABP system at different temperatures.

Formulation					Delayed crosslinking time (days)	Gel strength (mPa·s)	Initial viscosity (mPa·s)	Stability (days)
Salinity (mg/L)	Polymer P (mg/L)	Crosslinking agent A (%)	Coagulant B (%)	Temperature (°C)				
29500	3500	0.3	0.09	20	6-7	>11500	36.5	>60
				32	5-6	>14500	33	>60
				45	4-5	>16000	28.5	>60
				60	3-4	>17000	26.9	>60

**Table 4:** The plugging rates of the ABP system in different permeability cores.

Scheme	Cores number	Temperature (°C)	Permeability (mD)	*K*_1_ (mD)	*K*_2_ (mD)	Plugging rate (%)
1	C1	32	300	185	0	100
2	C2	32	500	291	0	100
3	C3	32	1000	595	2.11	99.7

**Table 5:** The results of oil displacement experiment in different plugging depth.

Profile control	Core permeable category	Plugging depth (cm)	Permeability (mD)	Average porosity (%)	Average saturation (%)	Recovery before plugging (%)	Recovery after plugging (%)	EOR by gel (%)	Total recovery (%)
No plugging	High	0	301.5	20.77	64.50	—	34.62	—	25.3
	Low		10.1	17.36	55.8	—	14.42	—	
The first plugging	High	10	300.8	20.45	64.55	34.62	44.3	9.68	34.39
	Low		10.3	17.33	55.57	14.42	22.81	8.39	
The second plugging	High	20	300.4	20.21	63.24	44.3	51.83	7.53	41.64
	Low		9.8	17.24	55.15	22.81	29.74	6.93	
The third plugging	High	30	303.3	21.66	65.71	51.83	53.2	1.37	45.07
	Low		10.1	17.52	56.68	29.74	35.58	5.84	
